# Genome-Wide Integration on Transcription Factors, Histone Acetylation and Gene Expression Reveals Genes Co-Regulated by Histone Modification Patterns

**DOI:** 10.1371/journal.pone.0022281

**Published:** 2011-07-29

**Authors:** Yayoi Natsume-Kitatani, Motoki Shiga, Hiroshi Mamitsuka

**Affiliations:** 1 Bioinformatics Center, Institute for Chemical Research, Kyoto University, Gokasho, Uji, Japan; 2 Institute for Bioinformatics Research and Development of Japan Science and Technology Agency (JST-BIRD), Saitama, Japan; Duke University, United States of America

## Abstract

N-terminal tails of H2A, H2B, H3 and H4 histone families are subjected to posttranslational modifications that take part in transcriptional regulation mechanisms, such as transcription factor binding and gene expression. Regulation mechanisms under control of histone modification are important but remain largely unclear, despite of emerging datasets for comprehensive analysis of histone modification. In this paper, we focus on what we call *genetic harmonious units (GHUs)*, which are co-occurring patterns among transcription factor binding, gene expression and histone modification. We present the first genome-wide approach that captures GHUs by combining ChIP-chip with microarray datasets from *Saccharomyces cerevisiae*. Our approach employs noise-robust soft clustering to select patterns which share the same preferences in transcription factor-binding, histone modification and gene expression, which are all currently implied to be closely correlated. The detected patterns are a well-studied acetylation of lysine 16 of H4 in glucose depletion as well as co-acetylation of five lysine residues of H3 with H4 Lys12 and H2A Lys7 responsible for ribosome biogenesis. Furthermore, our method further suggested the recognition of acetylated H4 Lys16 being crucial to histone acetyltransferase ESA1, whose essential role is still under controversy, from a microarray dataset on ESA1 and its bypass suppressor mutants. These results demonstrate that our approach allows us to provide clearer principles behind gene regulation mechanisms under histone modifications and detect GHUs further by applying to other microarray and ChIP-chip datasets. The source code of our method, which was implemented in MATLAB (http://www.mathworks.com/), is available from the supporting page for this paper: http://www.bic.kyoto-u.ac.jp/pathway/natsume/hm_detector.htm.

## Introduction

It is widely recognized that two sets of H2A, H2B, H3 and H4 histone families assemble to form an octamer, around which DNA wraps, turning in the condensation of DNA. Another important aspect of histone families is their N-terminal tails, which are subjected to posttranslational modifications such as methylation, acetylation, ubiquitination, ADP-ribosylation, and sumolation [1]. It is now recognized that these histone modifications are deeply involved in transcription regulation. For example, acetylation of lysine residues, which is one of the most well-investigated histone modifications, neutralizes the negative charge of DNA which results in the loosening of the wrapped DNA from a condensed, silenced state to an open active form [1]. Histone modifications are drawing attention also because it is closely related with disorders such as inflammation [2], diabetes [3], myelodysplasia [4], and cancer [5]. The efforts to develop new therapeutics using information on histone modifications have been rapidly growing [6].

Recent studies on histone modifications led to concrete and systematic understanding of its transcriptional regulation. Unique mechanisms of gene regulation by modified amino acid residues in histone are considered to be patterns, resulting in histone code hypothesis [7], [8]. Although this concept is under controversy, the importance of a comprehensive understanding of the histone modification is indicated by findings on the complicated molecular mechanisms under histone modifications. One example is a discovery that the preacetylated lysine 9 of histone H3 (H3 Lys9) works as a starting point to activate transcriptional elongation of *FOSL1* by triggering phosphorylation of H3 Ser10 by PIM1 kinase, acetylation of H4 Lys16 by histone acetyltransferase MOF, and association with BRD4 and CDK9 [9]. The motivation of this work is to find this type of relationships from large-scale datasets as patterns by detecting gene groups shared by expression, transcription factors and histone modification. We call these patterns *genetic harmonious units (GHUs)*.

For detecting GHUs, we need to consider many possible combinations between residues to be modified and TFs. For example, the number of possible combinations for *N* residues which can be modified reaches *2^N^*, and this large number of combinations makes detecting a histone modification pattern a difficult problem. This means that time- and cost-consuming traditional experimental methods of investigation are not necessarily best suited for finding histone modification patterns. Instead, powerful, high-throughput tools such as ChIP-chip (Chromatin Immunoprecipitation on chip) [10] perform more suitably to investigate histone modifications on a genome-wide basis. Nevertheless, regulation mechanisms in the GHUs remain largely unclear, despite that datasets for comprehensive analysis of histone modifications have already been accumulated in public repositories. Yuan et al. assessed the global regulatory role of histone acetylation by using publicly available ChIP-chip datasets and a simple regression method [11]. While they showed that multiple histone acetylation sites such as H3 Lys9 have cumulative regulatory effects on transcription rates, they concluded that “decoding the combinatorial complexity of histone modification requires not only new data but also new methods to analyze the data”. However, no methods have been presented since then, and we need to develop a methodology to find combinatorial histone modification patterns using large-scale biological datasets.

Informative datasets for the methodology can be suggested by [12], which checks whether interspecies differences in transcriptional regulation are directed by genetic sequence or nuclear environment by using Tc1 cells (hepatocytes derived from a mouse model of Down syndrome that contains human chromosome 21 in addition to the complete mouse genome). In terms of 1) TF-binding to DNA, 2) histone modification, and 3) gene expression, they found that patterns on human chromosome 21 in Tc1 cells matched those observed in human hepatocytes despite the nuclear environment of mouse hepatocytes, which indicates that genetic sequences are a major determinant factor of these three biological events [12]. This means that these events should be closely correlated with each other under given genetic sequence, which leads to the idea that GHUs could be obtained by extracting genes which share the same preferences in TF-binding, histone modification and gene expression. In fact, histone acetylation controls chromosome structure, which affects accessibility for TFs to DNA, and TFs controls transcription initiation, finally target genes being expressed. This also supports the idea that both certain types of histone acetylation and TFs regulate expression of target genes, which share the same biological function.

In light of the above, we develop a genome-wide and integrative approach for finding GHUs using datasets regarding TF-binding, histone modification and gene expression. Our approach employs ChIP-chip and microarray datasets with noise-robust soft clustering to systematically capture genes sharing patterns of TF-binding, histone modification and gene expression to detect GHUs. One clear advantage of our genome-wide, integrative, computational approach is that we can use a large number of data which are already experimented and accumulated.

## Methods

### Datasets

We used three different ChIP-chip datasets (matrices), all from yeast *Saccharomyces cerevisiae:* 1) ChIP-chip data for transcription factor (TR) with binding *t-*CDFs (Student' t cumulative distribution function) for 203 TFs as columns and 6,229 genes as rows [13], 2) ChIP-chip data for acetylated histones (AH) with binding intensities for 11 acetylated histones (H4 Lys8, H4 Lys12, H4 Lys16, H3 Lys9, H3 Lys14, H3 Lys18, H3 Lys23, H3 Lys27, H2A Lys7, H2B Lys11 and H2B Lys16) as columns and 2,453 genes as rows [14], and 3) ChIP-chip data for histones (HS) with binding intensities between two histones (four kinds of antibodies to H3 and three kinds of antibodies to H2B) as columns and 4,229 genes as rows [15]. In addition, we used two different gene expression datasets from NCBI' GEO (Gene Expression Omnibus) [16]: 1) Glucose depletion, GSE9217 [17] (GP: 12 experimental conditions for 5,716 genes) for evaluation of our method and 2) Histone acetyltransferase ESA1 mutant, GSE9840 (ES: eight conditions for 5,716 genes) for exploring new GHUs. We note that these two datasets can be retrieved from GEO under the condition that yeast cells were grown in 2% glucose YPD medium which is the same as the ChIP-chip datasets for TF-HM. All data are compliant with Minimal Information About Microarray Experiments (MIAME).

### Data normalization

To evaluate the acetylation strength over different histones, we normalized the intensity of each gene in AH by using the nucleosome occupancy in HS [15]. This manner was already used in [18], where the correlation between histone modification and TFs was detected. Our purpose is to capture the correlation among TFs, histone modification and gene expression. Thus we note that this normalization is well-suited for our purpose. Concretely, we first averaged the intensity of each gene in HS over the antibodies to each of H3 and H2B and then divided the intensity of each gene in AH by the averaged intensity of the corresponding gene and histone (Note that the averaged intensities in H3 and H2B are used for H4 and H2A, respectively. This is possible because the nucleosome core is formed of two H2A-H2B dimers and a H3-H4 tetramer [19], meaning that the occupancy of H2A should be approximately the same as that of H2B, and this is true of between H3 and H4.). We then constructed a new matrix AH+, with binding *t-*CDFs for 11 acetylated histones on 1,756 genes, which are shared between AH and HS.

### Clustering over ChIP-chip data

For each of TR and AH+, we grouped genes into *k* clusters, according to TF-binding and histone acetylation, respectively, by using spectral clustering [20], a standard clustering approach over a matrix in machine learning. Briefly, we performed the following: 1) Given a matrix 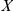, corresponding to TR (or AH+), we compute 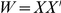, where 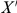 is the transpose of 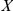, and the normalized graph Laplacian 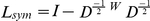, where 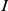 is the identity matrix and 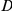 is the diagonal matrix in which the (*i*,*i*)-element 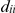 is given by 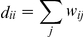, where 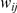
is the (*i*,*j*)-element of 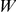. 2) We then compute eigenvectors of 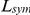 by eigen decomposition. 3) Using the first 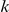 eigenvectors, we decide clusters over genes. A typical manner is hard clustering, such as *k*-means, which is rather noise-sensitive. We thus take soft clustering, more concretely a probabilistic model-based approach. That is, with the first 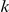 eigenvectors, we estimate parameters of a mixture of von Mises Fisher (vMF) distributions by using the Expectation-Maximization (EM) algorithm [21], resulting in soft clustering where genes are assigned clusters according to some confidence. The vMF distribution has the concentration parameter, κ, corresponding to the inverse of the variance. In terms of the stability and reproducibility of the cluster centroids, we set κ at ten by preliminary experiments, which will be described in the next section. We ran the EM algorithm 1,000 times with different initial values and used the result with the highest likelihood among 1,000 runs for further analysis.

We note that soft clustering allows to assign more than one clusters to a gene, and in particular, probabilistic model-based clustering including our approach allows to assign a cluster to a gene with a probability like that a gene can be in cluster 1 with probability of 0.8 and in cluster 2 with probability of 0.2. On the other hand, hard clustering such as *k*-means assigns only one cluster to a gene. This feature of soft clustering is reasonable and is a clear advantage, since in many cases one gene has multiple functions, which can be detected by soft clustering but not by hard assignment. This flexibility of soft clustering leads to an advantage in noise-robustness over hard clustering. We performed a preliminary experiment to check the stability and reproducibility of our approach by computing the variance of resultant cluster centroids, comparing with *k*-means, which was run in the same manner as our approach. The result was summarized into Table S2, which demonstrates that the variance of our approach is far smaller than that of *k*-means for all four datasets we used in our experiments, implying the noise-robustness of our approach, comparing with *k*-means. In addition, it was reported that applying soft clustering to microarray data analysis leads to more adequate clusters with information-rich structures, and increased noise-robustness [22].

### Parameter optimization

There are two parameters which we need to fix in our clustering method: the number of clusters (*k*) and the concentration parameter of the von-Mises Fisher distribution (κ). We fixed values of these parameters by a preliminary experiment: We changed these values (*k* = 5, 10 and 20, and κ = 5, 10 and 20), and for each setting of these values, we repeated running our clustering procedure (of 1,000 trials with different initial values and having a cluster set with the largest likelihood) three times and computed the variance of the centroid coordinates of resultant clusters over them to check the stability and reproducibility of resultant clusters. The result of this experiment is summarized into Table S1. Considering the smallest variances, we chose *k* = 10 and κ = 10 for AH+ (ChIP-chip for histone acetylation), while we chose *k* = 5 and κ = 10 for GP (microarray on glucose depletion) and ES (microarray on ESA1 mutant), where the variance was minimized (See the detail for Table S1). For TR (ChIP-chip for TFs), the minimum variance was obtained by *k* = 5, but this value was comparable with that by *k* = 10, and to make the matrix by TF and histone a balanced matrix, we chose *k* = 10 for TR. We used these parameter settings in our experiments.

### Selecting genes by using gene expression data

We generated a TF-histone matrix (TF-HM) by using ten clusters from TR and ten clusters from AH+. This is simply done by assigning each of 1,756 genes (shared between TR and AH+) to one of 10×10 clusters, resulting in that all 1,756 genes are partitioned into 100 clusters. At the same time we generated five gene clusters from each of GP and ES (both of which include 1,730 genes shared with TF-HM) by using our clustering approach. We then generated 500 ( = 5×10×10) clusters of 1,730 genes by using the five clusters generated and TF-HM. That is, we assigned each gene to one of 500 clusters, resulting in that all genes were partitioned into 500 clusters. We counted the number of genes which were assigned to each element of 500 clusters. Then, the number of genes in each element was converted into a *t-*CDF, and we chose elements with *t-*CDFs of more than 0.99 (0.99 was chosen to keep the number of elements approximately 15, which would be the maximum number for which we could check the detail of genes in the elements manually, e.g. for GP, 14 elements with *t*-CDFs of more than 0.99 while 25 with 0.95, and for ES, 14 with 0.99 while 30 with 0.95). We further checked the *p*-value of MIPS functions (See the next section) in selected elements and elements with MIPS functions of *p*<0.01 were selected and referred to as *pattern-elements* for further analysis.

### Characterizing clusters with MIPS functions

We characterized each cluster by checking the MIPS functions of genes for each element by using FunCat (http://mips.helmholtz-muenchen.de/projects/funcat)[23], which gives a *p*-value showing the probability that genes in a cluster have the corresponding MIPS function against the null hypothesis that these genes are randomly selected. Throughout this work, 0.01 was chosen for the cut-off *p*-value to keep the number of overrepresented MIPS functions at a moderate size, e.g. for ten clusters from TR, 26 MIPS functions overrepresented by *p*<0.01 while 87 by *p*<0.05, for ten clusters from AH+, 45 MIPS functions overrepresented by *p*<0.01 while 85 by *p*<0.05, for five clusters from GP, 29 MIPS functions overrepresented by *p*<0.01 while 66 by *p*<0.05, and for five clusters from ES, 44 MIPS functions overrepresented by *p*<0.01 while 79 by *p*<0.05. We note that 0.01 was used for selecting pattern elements in the final step of our procedure.

### Extracting patterns in pattern-elements

1) TF-binding: We calculated TF-scores to select TFs which bind to promoter regions of genes in each of the selected elements. For each of GP and ES, we computed the following TF-score from TR:



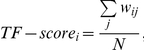
where 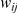 is the (*i*,*j*)-element of TR (*i* is for TF and *j* is for genes) and *N* is the number of genes in the corresponding pattern-element. We then extracted TFs which are closely related with each pattern-element, by selecting TFs with TF-scores of more than 0.8 ( = 0.9×0.9), which is equivalent to selecting TFs which bind to the promoter regions of 90% of genes in one selected element with probability 90%.

2) Histone acetylation: See the section “Statistical testing” below.

3) Gene expression: We presented the patterns of gene expressions for each pattern-element, along with their averages.

### Statistical testing: SDAL (Statistically detecting pattern-elements with Differentially Acetylated Lysine residues) testing

To find a pair of differentially acetylated lysine residues, we adopted a two-step procedure: We first used ANOVA (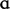 = 0.01), and for elements which pass the ANOVA testing, we further used the Tukey-Kramer' multiple comparison test (*p*-value <0.05). We ran this two-step procedure over all pairs of 11 lysine residues to check whether each lysine residue of histones which are related to genes in each element is significantly acetylated. This two-step procedure is used to further focus on important pattern-elements. In this test, we used only the corresponding genes in AH+ for each pattern-element.

### Comparison with randomized datasets

We conducted a comparative experiment by using randomized datasets: We first generated randomized datasets from TR, AH+, GP and ES by shuffling both rows and columns of the corresponding table. We then run the same procedure as that of our method over these randomized datasets, resulting in that we had elements with high *t*-CDFs of more than 0.99 and having MIPS functions with *p*-values. These two steps were repeated *N_rand_* times, by which we had a set of *p*-values. We used Mann Whitney U-test under the null hypothesis that the distribution of *p*-values from randomized datasets and that from TR, AH+, GP (or ES) are the same (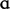 = 0.01).

### Accession numbers

The GenBank (http://www.ncbi.nlm.nih.gov/Genbank/) accession numbers for genes and proteins discussed in this paper are ARP4 (NP_012454), BRD4 (NP_490597), CDK9 (NP_001252), ESA1 (NP_014887), FHL1 (NP_015429), *FOSL1* (NP_005429), HOG1 (NP_013214), *HXT1* (NP_011962), *HXT2* (NP_013724), *HXT3* (NP_010632), *HXT4* (NP_011960), MIG2 (NP_011306), MOF (NP_115564), MSN2 (NP_013751), MSN4 (NP_012861), MTH1 (NP_010563), PIM1 (NP_002639), RAP1 (NP_014183), RGT2 (NP_010143), SIR2 (NP_010242), SNF3 (NP_010087) and SUP2 (NP_010457).

### Implementation

The source code of our method, which was implemented in MATLAB (http://www.mathworks.com/), is available from the supporting page for this paper: http://www.bic.kyoto-u.ac.jp/pathway/natsume/hm_detector.htm.

## Results and Discussion

### GHUs responsible for adaptation to glucose depletion

**Figure 1:**
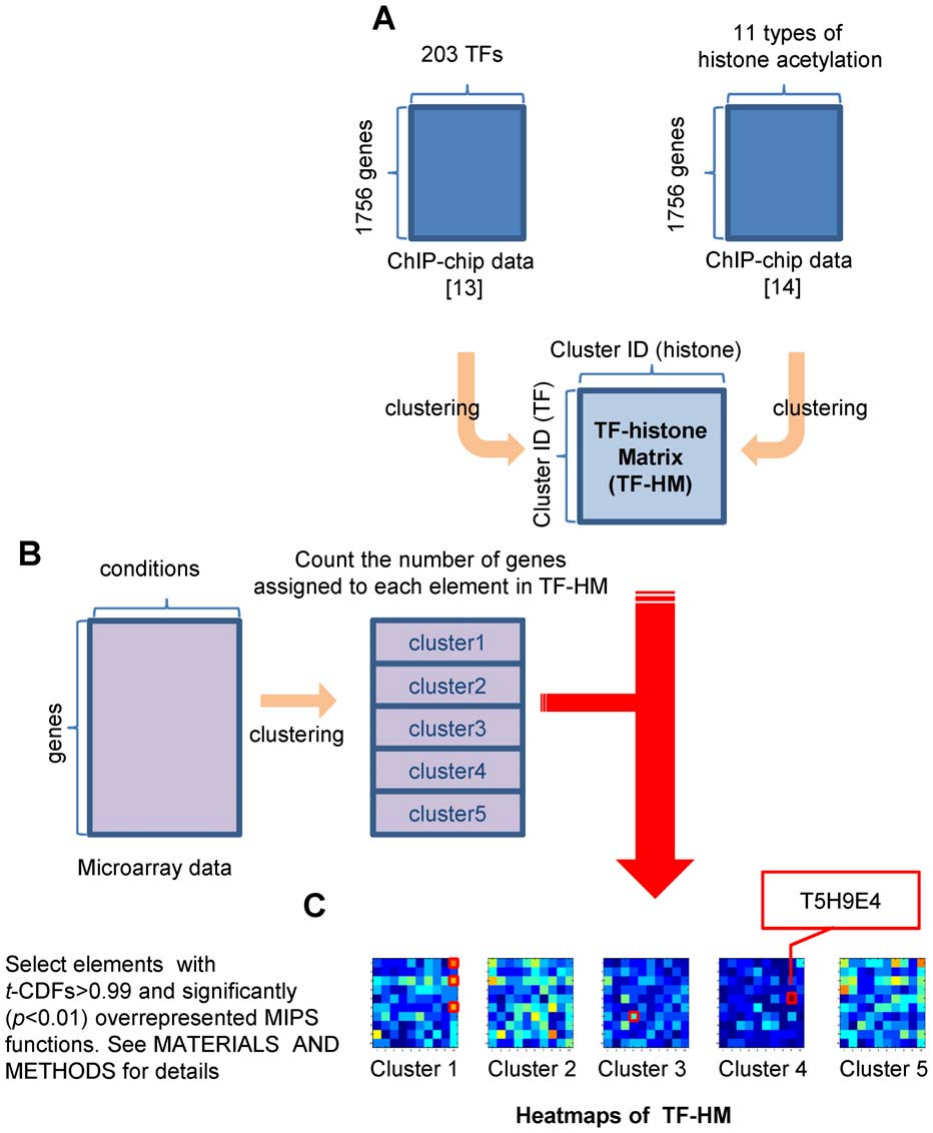
A schematic overview of the proposed approach. (A) Preparation of the TF-HM matrix using the clustering results of ChIP-chip datasets. This matrix has cluster IDs for TF-binding as one dimension and cluster IDs for histone acetylation as the other dimension. (B) Clustering genes with microarray data into five groups to generate 500 clusters by using TF-HM. We then compute the number of genes assigned to each of 500 clusters which is turned into *t*-CDF. (C) Heatmaps of 500 clusters (or five TF-HMs). Out of 500 ( = 5×10×10) possible elements, we select those with *t-*CDFs of more than 0.99 and overrepresented MIPS functions (*p*<0.01). These chosen elements are used to detect patterns of the GHUs, in terms of histone acetylation as well as TF-binding and gene expression. Each of 500 clusters is named like T5H9E4, standing for cluster 5 of TR, cluster 9 of AH+ and cluster 4 of gene expression. See main text for details.

We used two ChIP-chip datasets (TR and AH+) in yeast [13], [14]. Our method first generated gene clusters in terms of TF-binding and those in terms of histone acetylation, from TR and AH+, respectively (The number of clusters is set at ten; See MATERIALS AND METHODS). The biological functions of the generated clusters were checked by characterizing each cluster using significantly (*p*<0.01) overrepresented functional categories (which we call MIPS functions; Table S3 for TR and Table S4 for AH+) of FunCat [22]. We then generated a 10×10 matrix (or table, which we call TF-HM) where in each matrix cell (or element), genes in the associated cluster behave similarly according to both TF-binding and histone acetylation (Fig. 1A).

**Figure 2:**
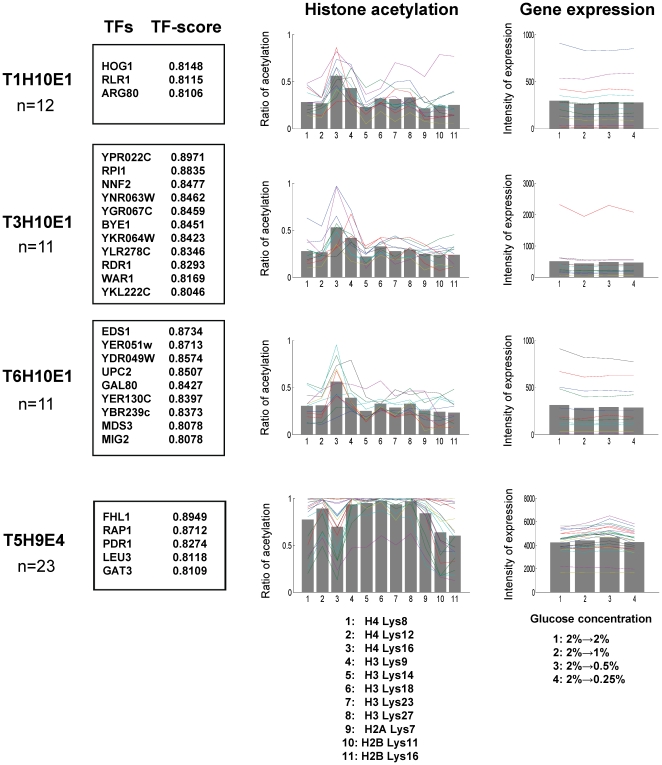
The GHUs obtained from microarray data regarding glucose depletion. The detected patterns of TF-binding, histone acetylation and gene expression. Each row corresponds to a pattern-element, which is labeled by using cluster IDs of TF-binding, histone acetylation and gene expression (e.g. T1H10E1: cluster 1 of the TF-binding clusters, cluster 10 of the histone acetylation clusters, and cluster 1 of the gene expression clusters), and the number of genes (e.g. n = 12 means that 12 genes for T1H10E1). For each row, we showed TFs with TF-scores of more than a pre-specified threshold (0.8), acetylation rates of 11 lysine positions and the variation in gene expression by changing glucose concentration. Each line in graphs corresponds to a gene, and each bar shows the average over the corresponding values.

Our method further grouped genes in terms of expression patterns by subdividing genes in each element of TF-HM according to gene expression. Here we used a microarray dataset on a well-studied issue of histone acetylation, i.e. glucose depletion (GSE9217). We partitioned genes in this microarray dataset (1%, 0.5% and 0.25% glucose YPD media against control of 2%) into five clusters (Fig. 1B) and checked the MIPS functions which were significantly (*p*<0.01) overrepresented in each cluster (Table S5). For each of the five clusters, we checked the number of genes which were assigned to each element of TF-HM (Fig. 1C). Out of the total 500 ( = 5×10×10) possible elements, 14 elements with *t*-CDFs (Student' t cumulative distribution function) of more than 0.99 (*t*>0.99) were first selected (Figs. S1A and S1B). Out of the 14 elements, we then selected four pattern-elements which have significantly (*p*<0.01) overrepresented MIPS functions (See MATERIALS AND METHODS) and pass SDAL testing for lysine residue pairs (Fig. 2).

**Figure 3:**
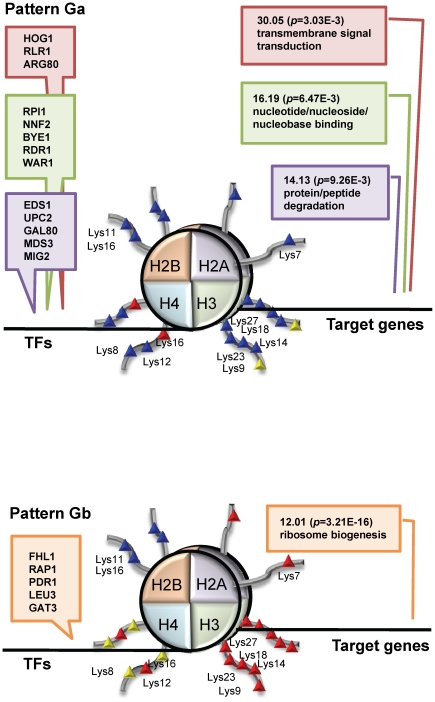
A schematic diagram of the detected two GHUs in Fig. 2. Pattern Ga: T1H10E1, T3H10E1 and T6H10E1 are put together into pattern Ga, because they are regulated by the same acetylated residues of histone and show the same variation in gene expression intensities. The list boxes on TFs and MIPS functions of target genes are represented by three colors corresponding to three pattern elements: red square: T1H10E1, green square: T3H10E1 and purple square: T6H10E1. For example, T1H10E1 shows that TFs (HOG1, RLR1 and ARG80) in the red box regulate genes related with the MIPS function in the red box (transmembrane signal transduction) in an acetylated H4 Lys16-dependent manner. Only annotated TFs are shown. Pattern Gb: obtained from T5H9E4. In each row, boxes on the left-hand side represent frequently-binding TFs, and those on the right-hand side represent the overrepresented MIPS functions in each GHU (*p*<0.01). The two overlapping circles in each row represent a histone octamer with its N-terminal tails and lysine residues (▴). The number attached to each ▴ is the corresponding residue position. The colors of the lysine residues reflect the results from a multiple comparison test (See Table S6 for the entire result of the test): Red ▴ shows significantly acetylated lysine residues against those represented by blue ▴ (*p*<0.05), and yellow ▴ shows other lysine residues.

Out of four pattern-elements obtained (T1H10E1, T3H10E1, T6H10E1 and T5H9E4), T1H10E1, T3H10E1 and T6H10E1 shared the same acetylated residues of histone (H4 Lys16) and variation in expression intensities of target genes, although TFs are different. We then put these three pattern-elements together as pattern Ga in Fig. 3, implying that one type of histone acetylation regulates three types of clusters of TFs, which results in only one type of gene expression. Thus we have two different patterns (Fig. 3: patterns Ga (T1H10E1, T3H10E1 and T6H10E1) and Gb (T5H9E4)), where we colored lysine residues red if significantly (*p*<0.05) acetylated by a multiple comparison test in SDAL testing (Table S6).

Under glucose depletion, yeast cells switch the energy-supplying reaction from fermentation to respiration. This change results in the elevation of cellular NAD^+^ concentration, which in turn activates the class III HDAC SIR2, causes NAD^+^-dependent deacetylation of H4 Lys16 and invokes generalized gene inactivation by chromatin silencing [24]. In other words, H4 Lys16 must be kept acetylated in the steady state, where glucose depletion does not happen. In fact, AH and HS are expected to be obtained under the steady state (where glucose depeletion does not happen), and Fig. 2 shows H4 Lys16 is well acetylated, implying the consistency between our result and the expectation from the literature. In pattern Ga where only H4 Lys16 is colored red, expression intensities of target genes were unchanged for all three pattern elements, meaning that these genes were inactivated both at the steady state (1: 2% ->2%) and under glucose depletion (2, 3 and 4: 2% ->1, 0.5 and 0.25%, respectively). This result indicates that acetylated H4 Lys16 might be deeply involved with the regulation of TFs in pattern Ga to keep their target genes inactivated, which implies the validity of pattern Ga. Furthermore, HOG1, a TF in pattern Ga, interacts with glucose-regulated transcription factor MSN2/MSN4 [25]–[26]. MIG2, another TF in pattern Ga, is a glucose-regulated TF and represses genes involved in metabolism of alternative carbon sources such as galactose (*GAL* genes) and maltose (*MAL* genes) under high concentration of glucose [25]. This result also supports pattern Ga, implying that some TFs which are already known to work under high concentration of glucose might remain inactivated in pattern Ga.

In pattern Gb, genes related to ribosome biogenesis were up-regulated independently of H4 Lys16. More concretely, target genes were up-regulated under mildly low concentration of glucose (2 and 3: 2% ->1% and 0.5%, respectively) but not necessarily under extremely low concentration (4: 2% ->0.25%). Ribosome biogenesis is responsible for global stress response [27], which thus may not be glucose depletion-specific. Furthermore this result is consistent with a report that genes involved in cytoplasmic ribosomes respond only to glucose concentrations of >0.1% and both transcriptional and post-transcriptional mechanisms combine to accelerate the accumulation of ribosomal protein mRNAs [28]. Among the selected TFs in pattern Gb, FHL1 [29] and RAP1 [27] are independently reported to regulate ribosome biogenesis and were clearly dependent on the same type of histone acetylation (co-acetylation of five lysine residues in H3 with H4 Lys12 and H2A Lys7), which also supports pattern Gb.

Overall, two GHUs we detected can be characterized by: i) generalized gene inactivation caused by deacetylation of H4 Lys16 and ii) up-regulation of genes related to ribosomal biogenesis. These two patterns have supportive literature, validating the results as well as our approach for finding GHUs.

We further performed comparison with randomized datasets, keeping *N_rand_* = 10, and confirmed that the distribution of *p*-values on MIPS functions was significantly different from that of randomized datasets (*p*-value: 0.0078<0.01).

### GHUs responsible for the essential role of ESA1

**Figure 4:**
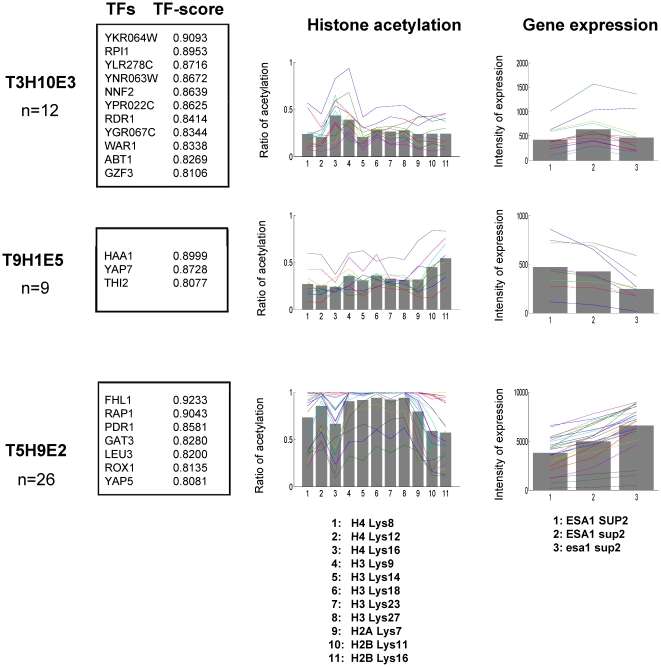
The GHUs obtained from microarray data for ESA1 and/or SUP2 mutants. The detected patterns of TF-binding, histone acetylation and gene expression are shown in the same manner as in Fig. 2: Each row is a pattern-element labeled with the cluster ID obtained from TF-binding, histone acetylation and gene expression.

**Figure 5:**
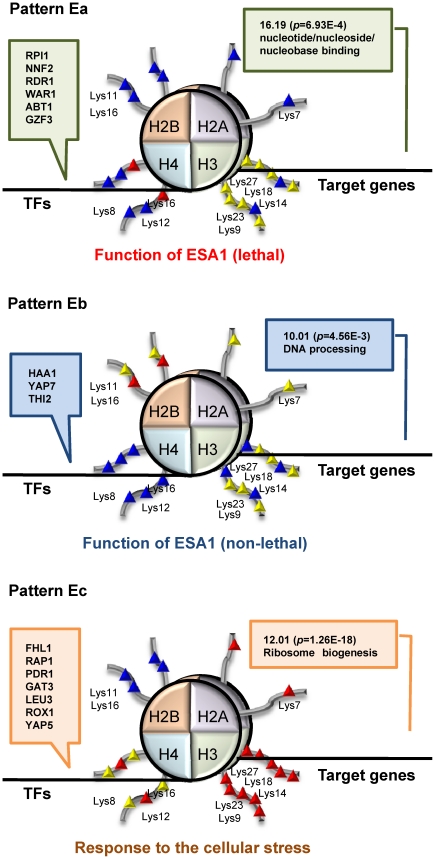
A schematic diagram of the detected GHUs in Fig. 4. Pattern Ea: obtained from T3H10E3. Only annotated TFs are shown. Pattern Eb: obtained from T9H1E5. Pattern Ec: obtained from T5H9E2. Each pattern is shown in the same manner as that in Fig. 3. The entire result of the multiple comparison test, by which lysine residues are colored, is shown in Table S8.

Here we replaced the microarray dataset (GSE9217) with another dataset for ESA1 mutant (GSE9840). We again partitioned genes into five clusters, for which we checked associated MIPS functions, which were all significantly (*p*<0.01) overrepresented (Table S7). Then, out of total 500 ( = 5×10×10) possible elements, 11 elements (*t*>0.99) were first selected (Figs. S2A and S2B). Out of the 11 elements, three pattern-elements which have significantly (*p*<0.01) overrepresented MIPS functions and pass SDAL testing for lysine residue pairs were further selected, resulting in three kinds of GHUs in TF-binding, histone acetylation and gene expression (Fig. 4 and Fig. 5: patterns Ea (T3H10E3), Eb (T9H1E5) and Ec (T5H9E2)). We again colored lysine residues in red (Fig. 5) if significantly (*p*<0.05) acetylated according to a multiple comparison test (Table S8).

**Figure 6:**
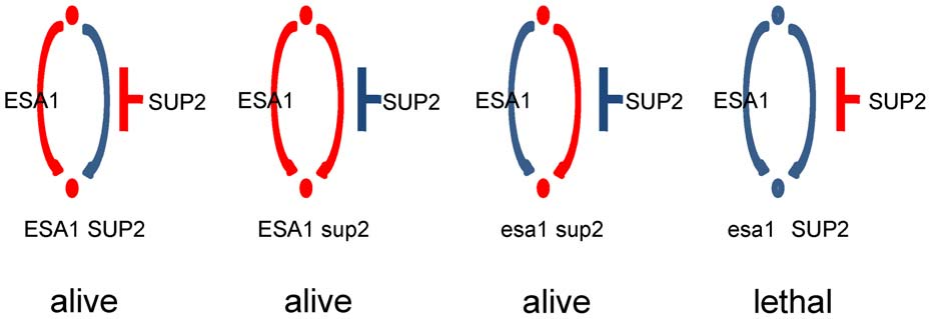
Schematic pathways presumed for the essential function of ESA1. Red path: intact, and blue path: damaged. The ESA1 sup2 mutant is supposed to transmit a signal excessively, while the esa1 SUP2 mutant fails to transmit a signal downstream.

ESA1 is an essential histone acetyltransferase (HAT), which acetylates primarily histone H4 [30], [31]. In spite of its well-known HAT activity, the essential role of ESA1 is under controversy. It is reported that the essential function of ESA1 may be to bind acetyl-CoA or lysine substrates, not to function as HAT, because single mutations in the catalytic pocket of ESA1 (with loss of catalytic activity of ESA1) were not lethal [32]. To specify the essential role of ESA1, a bypass suppressor of ESA1 (SUP2) was identified by bypass suppression screening for GSE9840 dataset. We attempted to characterize the role of ESA1 by comparing the gene expression profiles of wild type (ESA1 SUP2), SUP2 mutant (ESA1 sup2) and ESA1 SUP2 double mutant (esa1 sup2). We expected that expression intensities of genes related to the essential function of ESA1 should change in ESA1 sup2 mutant, when compared to ESA1 SUP2 and esa1 sup2 (Fig. 6). In fact this was shown in pattern Ea (Fig. 5), indicating that the genes of interest were assigned to pattern Ea. In pattern Ea, genes were dependent on acetylated H4 Lys16 only (Fig. 5), meaning that the recognition of acetylated H4 Lys16 would be crucial for ESA1. This result is consistent with the literature which suggests the involvement of acetyl-CoA or lysine substrates [32], and implying more detail: the involvement of acetylated H4 Lys16. H4 Lys16 is not a compatible substrate of ESA1 in spite of its preference for H4 [33]. This implies that H4 Lys16 is distinguished from other lysine residues in H4, and serves as the basis for ESA1 to recognize H4. Meanwhile, the gene expression of pattern Eb indicates that ESA1 mutation rather than SUP2 mutation was responsible for gene regulation, although this function was not essential. In pattern Eb, genes are dependent on acetylated H2B Lys16, which might also be recognized by ESA1 for a different response. Both patterns Ea and Eb include genes such as DNA helicase and mitochondrial transporter (Fig. S2B), demonstrating that ESA1 controls DNA turnover and mitochondrial functions under the corresponding histone acetylation pattern. In fact, the acetylation of histone H4 is related to the recruitment of ESA1-ARP4 HAT complex and is required for DNA double-strand break repair [34]. Finally, we found that pattern Ec is consistent with pattern Gc in TF-binding, histone acetylation and overrepresented MIPS functions. Pattern Ec shows that genes were up-regulated equally by each of two mutations. This implies that the number of mutations rather than gene specificity was influential on gene expression, demonstrating that genes regarding ribosome biogenesis were up-regulated in response to the cellular stress caused by gene mutations. In addition, we performed comparison with randomized datasets, keeping *N_rand_* = 10 again, and confirmed that the distribution of *p*-values on MIPS functions was significantly different from that of randomized datasets (*p*-value: 0.0015<0.005).

### Conclusion

We have developed a genome-wide and data-driven method, which discovers patterns of histone acetylation through correlations with TF-binding and gene expression. This is the first genome-wide approach of integrating three types of information, i.e. TF-binding, histone acetylation and gene expression, for detecting GHUs. In addition to the report that inspired this work [12], both the correlations between histone acetylation and TF-binding [18], [35] and between TF-binding and gene expression [13] have been already reported, implying that our integration is reasonable because our approach is an expansion along with the literature in terms that all three types of data are used. We stress that our method revealed a well-studied histone modification pattern in a GHU under glucose depletion as well as new patterns related with ESA1 functions, which would be valid in terms of the latest literature.

By using a microarray dataset measured under another experimental condition, our method might find unknown GHUs which are obtained under the given condition. In other words, experimentalists can use any microarray dataset as an input of our method to learn histone modification patterns under the condition of interest. Similarly, we may find other patterns by considering different modifications, such as methylation and phosphorylation, by using a ChIP-chip dataset for methylated or phosphorylated histones.

## Supporting Information

Figure S1 **Elements with** ***t-*** **CDFs of more than 0.99 (GSE9217).** (A) Each of five heatmaps represents the number of genes assigned to each element in TF-HM under the corresponding one of five clusters from GSE9217. Red square: elements with *t-*CDFs>0.99. (B) List of genes in each of elements with *t*-CDFs of more than 0.99. Each element ID consists of cluster IDs of TF-binding, histone acetylation and gene expression (e.g. T9H1E1: cluster 9 of the TF-binding clusters, cluster 1 of the histone acetylation clusters, and cluster 1 of the gene expression clusters).
(PDF)

Figure S2 **Elements with** ***t-*** **CDFs of more than 0.99 (GSE9840).** (A) Each of five heatmaps represents the number of genes assigned to each element in TF-HM under the corresponding one of five clusters from GSE9840. Red square: elements with *t-*CDFs>0.99. (B) List of genes in each of elements with *t*-CDFs of more than 0.99. Each element ID consists of cluster IDs of TF-binding, histone acetylation and gene expression (e.g. T3H1E1: cluster 3 of the TF-binding clusters, cluster 1 of the histone acetylation clusters, and cluster 1 of the gene expression clusters). DNA helicases are colored pink. Mitochondrial transporters are colored green.
(PDF)

Table S1 Results of preliminary experiment: Variances of the coordinates of cluster centroids obtained by clustering of genes in datasets TR, AH+, GP and ES. To optimize two parameters *k* and κ in our approach, we repeated our clustering procedure (which runs our clustering algorithm 1,000 trials with different initial values and obtains a set of clusters which gives the largest likelihood out of 1,000 trials) three times and computed the variance of the coordinates of cluster centroids over three runs. The smallest values are in boldface. A) Optimization of concentration parameter κ. The results by κ = 10 were more stable and reproducible (the variance is the smallest) than those by κ = 5 or 20 for both TR and AH+. We chose κ = 10 in our experiments. B) Optimization of the number of clusters *k*. The smallest variance made us chose *k* = 10 for AH+, and *k* = 5 for GP and ES. For TR, the result by *k* = 10 was comparable with that by *k* = 5, and we chose *k* = 10, making TF-HM (TF-histone matrix) a balanced matrix.
(DOC)

Table S2 Results of preliminary experiment: Variances of the coordinates of cluster centroids obtained by clustering of genes in datasets TR, AH+, GP and ES. We used the parameter set fixed in Table S1 for our approach. Also the variance of our method was obtained by the same manner as that of Table S1. On the other hand, the variance of *k*-means was obtained in the same manner. That is, we repeated the following process three times: we run *k*-means 1,000 times with random initial values and obtain the best cluster sets, and computed the variance of the coordinates of cluster centroids over resultant three cluster sets. The smallest value for each dataset is in boldface. This result clearly shows the advantage of our approach over *k*-means in reproducibility and stability of resultant clusters.
(DOC)

Table S3 **Overrepresented MIPS functions in ChIP-chip data** [**13**] **.** We show Level 1 and 2 of MIPS functions only. *P*-values represent the probability of finding the observed number of genes with the specified MIPS function under the null hypothesis that the genes were selected at random.
(DOC)

Table S4 **Overrepresented MIPS functions in ChIP-chip data** [**14**] **.** We show Level 1 and 2 of MIPS functions only. *P*-values represent the probability of finding the observed number of genes with the specified MIPS function under the null hypothesis that the genes were selected at random.
(DOC)

Table S5 **Overrepresented MIPS functions in microarray data (GSE9217).** We show Level 1 and 2 of MIPS functions only. *P*-values represent the probability of finding the observed number of genes with the specified MIPS function under the null hypothesis that the genes were selected at random.
(DOC)

Table S6 **The result of Tukey-Kramer' multiple comparison test (GSE9217,** ***p*** **<0.05).** The lysine residue pairs which were significantly different from each other are listed.
(DOC)

Table S7 **Overrepresented MIPS functions in microarray data (GSE9840).** We show Level 1 and 2 of MIPS functions only. *P*-values represent the probability of finding the observed number of genes with the specified MIPS function under the null hypothesis that the genes were selected at random.
(DOC)

Table S8 **The result of Tukey-Kramer' multiple comparison test (GSE9840,** ***p*** **<0.05).** The lysine residue pairs which were significantly different from each other are listed.
(DOC)
