# Supplementary material for: Genome-Wide Integration on Transcription Factors, Histone Acetylation and Gene Expression Reveals Genes Co-Regulated by Histone Modification Patterns
Source: PLoS One. 2011 Jul 29;6(7):e22281. doi: 10.1371/journal.pone.0022281 (PMC3146477; doi:10.1371/journal.pone.0022281)
Supplement: Figure S1 — Elements with t- CDFs of more than 0.99 (GSE9217). (A) Each of five heatmaps represents the number of genes assigned to each element in TF-HM under the corresponding one of five clusters from GSE9217. Red square: elements with t-CDFs>0.99. (B) List of genes in each of elements with t-CDFs of more than 0.99. Each element ID consists of cluster IDs of TF-binding, histone acetylation and gene expression (e.g. T9H1E1: cluster 9 of the TF-binding clusters, cluster 1 of the histone acetylation clusters, and cluster 1 of the gene expression clusters). (PDF) [file pone.0022281.s001.pdf]

A

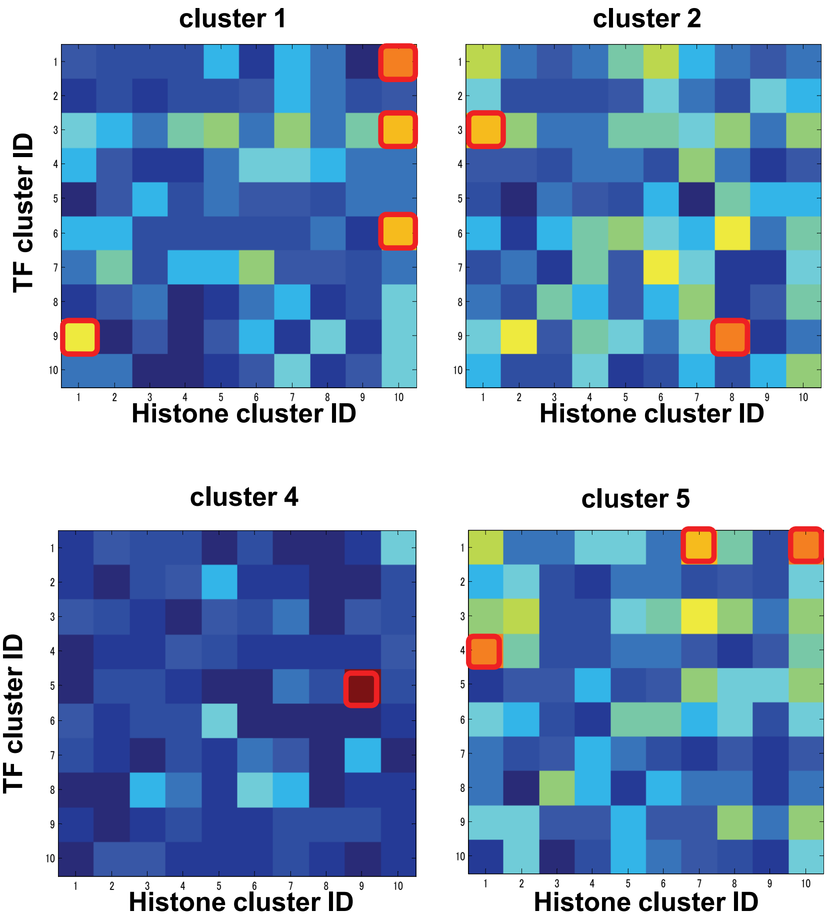

B

element ID	*t*-CDF	genes
T9H1E1	0.9939	*VAM6, RAD34, YHR182W, FYV10, REC107, ACF4, HCS1, PRR1, IMP1, MDL2*
T1H10E1	0.9994	*VPS8, STE5, PAM1, TGL1, PXA2, YNR065C, WSC3, HRP1, KIN4, YOR387C, CHL1, KAP120*
T3H10E1	0.9981	*BEM1, DUG2, DOP1, PBS2, VPS13, PIG1, VBA1, PMS1, CRT10, ALA1, MCM4*
T6H10E1	0.9981	*CDC53, YDL180W, DPL1, CDC20, IME4, PDC6, DSE4, TRE2, ISW2, SOG2, NTO1*
T3H1E2	0.9922	*PER1, MCH1, YDR026C, GDA1, CSM2, MDE1, SKG3, MRPS17, DSS1, MSB4, RRI2*
T9H8E2	0.9973	*GPI8, IRC3, APC2, ATG17, DML1, HER2, VAC7, PHA2, COQ10, ATX2, YOR111W, LIP5*
T3H1E3	0.9982	*AKR1, ATP22, PIC2, PET54, AIM24, YKL162C, PEX1, GAD1*
T5H3E3	0.9982	*TGL2, YDR065W, IPT1, UBP1, CTR2, CBT1, ARA2, GDB1*
T8H3E3	0.9982	*AMN1, YGR250C, HXT5, MSN4, YLR312C, YNL144C, YGP1, SLA2*
T7H4E3	0.9911	*PAT1, COX20, GSY1, SMF2, AIM46, GUT2, YPS1*
T5H9E4	1.0000	*RPS8A, RPS13, ARB1, RPL34A, RPS24A, RPL11B, RPL42B, RPS4B, RPL39, RPL17A, RPS17A, RPS18B, PHO84, SSB2, RPL6A, PRE6, RPL33B, RPL21B, RPL5*
T4H1E5	0.9975	*FUN26, LRE1, YDR161W, YDR514C, PAD1, YHL039W, EPS1, TAF4, BNI4, YNL295W, CLP1, SSO1*
T1H7E5	0.9928	*PHO87, TMA20, HOM3, SWP82, ARD1, ARG4, HCA4, PRS1, YSH1, ERB1, SRP72*
T1H10E5	0.9975	*ISW1, ENP1, RAD3, YGL114W, QNS1, SKI2, WAR1, PIK1, RPO31, UBP2, ADE2, FDH1*

Natsume-Kitatani et al., Fig. S1
