# Supplementary material for: Genome-Wide Integration on Transcription Factors, Histone Acetylation and Gene Expression Reveals Genes Co-Regulated by Histone Modification Patterns
Source: PLoS One. 2011 Jul 29;6(7):e22281. doi: 10.1371/journal.pone.0022281 (PMC3146477; doi:10.1371/journal.pone.0022281)
Supplement: Figure S2 — Elements with t- CDFs of more than 0.99 (GSE9840). (A) Each of five heatmaps represents the number of genes assigned to each element in TF-HM under the corresponding one of five clusters from GSE9840. Red square: elements with t-CDFs>0.99. (B) List of genes in each of elements with t-CDFs of more than 0.99. Each element ID consists of cluster IDs of TF-binding, histone acetylation and gene expression (e.g. T3H1E1: cluster 3 of the TF-binding clusters, cluster 1 of the histone acetylation clusters, and cluster 1 of the gene expression clusters). DNA helicases are colored pink. Mitochondrial transporters are colored green. (PDF) [file pone.0022281.s002.pdf]

A

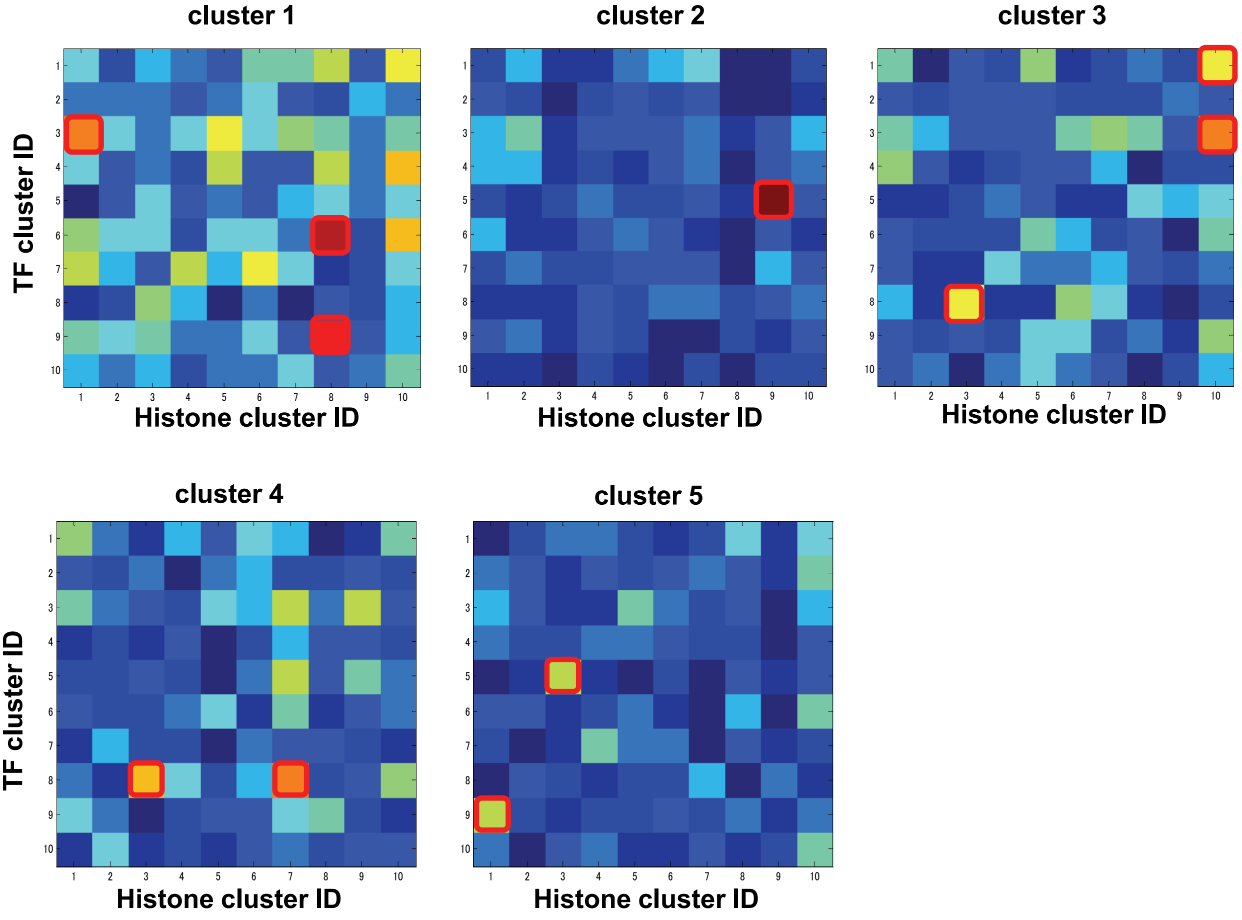

B

element ID	*t*-CDF	*genes*
T3H1E1	0.9913	*AIM2, ATP22, PET54, HSE1, MDE1, AIM24, MNR2, NTR2, CHA4, GAD1, RRI2, PTC5*
T6H8E1	0.9996	*YBP1, PLP1, FMN1, YDR282C, MRPL35, CIN8, YFH7, GTO1, PET130, YKR017C, HRT3, RNH1, NGL1, PHR1, LEA1*
T9H8E1	0.9988	*IRC3, LRS4, KRE29, SAY1, YIL152W, YIR024C, TTI2, APC2, ATG17, CSI1, VAC7, YOR111W, APC5, PNT1*
T5H9E2	1.0000	*RPS8A, RPL13A, RPS13, HPT1, RPL34A, RPS24A, RPL11B, RPL42B, RPS4B, RPL39, SFT1, RPL17A, RPS17A, RPS18B, RPL6A, PHO84, SEC59, SSB2, NOG2, RPL33B, RPL21B, RPL5*
T8H3E3	0.9951	*AMN1, SOR2, MIG3, YER158C, HSP150, HXT9, CLN1, SLA2, TRM13*
T1H10E3	0.9951	*ISW1, PCA1, SKI2, YNR065C, WSC3, KIN4, YOR387C, FDH1, CHL1, HOS3*
T3H10E3	0.9996	*DUG2, POL3, SAP1, HOP2, YJR030C, YJR030C, YJR061W, PIG1, STE23, VBA1, ATM1, MCM4, AGC1*
T8H3E4	0.9986	*YBR238C, TOS3, MGA1, PCL5, VHR1, MSN4, SOK2, PFK2, AQR1, PDR5, YOR342C*
T8H7E4	0.9996	*PGI1, BAP3, PTR2, HOG1, YLR460C, MSN2, MCM1, ILV2, ZWF1, PFK27, ISU2, CLB2*
T9H1E5	0.9989	*RAD34, RNH70, YHR131C, YHR182W, REC107, HCS1, PET494, MDL2, GRS2*
T5H3E5	0.9989	*DIA3, TGL2, YDR065W, RAD7, CBT1, FBP1, YNL035C, LPE10, GDB1*

DNA helicase, mitochondrial transporter

Natsume-Kitatani et al., Fig. S2
