# Supplementary material for: Genome-Wide Integration on Transcription Factors, Histone Acetylation and Gene Expression Reveals Genes Co-Regulated by Histone Modification Patterns
Source: PLoS One. 2011 Jul 29;6(7):e22281. doi: 10.1371/journal.pone.0022281 (PMC3146477; doi:10.1371/journal.pone.0022281)
Supplement: Table S1 — Results of preliminary experiment: Variances of the coordinates of cluster centroids obtained by clustering of genes in datasets TR, AH+, GP and ES. To optimize two parameters k and κ in our approach, we repeated our clustering procedure (which runs our clustering algorithm 1,000 trials with different initial values and obtains a set of clusters which gives the largest likelihood out of 1,000 trials) three times and computed the variance of the coordinates of cluster centroids over three runs. The smallest values are in boldface. A) Optimization of concentration parameter κ. The results by κ = 10 were more stable and reproducible (the variance is the smallest) than those by κ = 5 or 20 for both TR and AH+. We chose κ = 10 in our experiments. B) Optimization of the number of clusters k. The smallest variance made us chose k = 10 for AH+, and k = 5 for GP and ES. For TR, the result by k = 10 was comparable with that by k = 5, and we chose k = 10, making TF-HM (TF-histone matrix) a balanced matrix. (DOC) [file pone.0022281.s003.doc]

**Natsume-Kitatani et al., Table S1**

|  |  |  |  |  |  |
| --- | --- | --- | --- | --- | --- |
|  | **A** | κ=5 | κ=10 | κ=20 |  |
|  | TR (*k*=10) | 8.15E-04 | **2.62E-32** | 3.1781 |  |
|  | AH+ (*k*=10) | 4.741 | **2.64E-32** | 5.7323 |  |
|  |  |  |  |  |  |
|  |  |  |  |  |  |
|  | **B** | *k*=5 | *k*=10 | *k*=20 |  |
|  | TR (κ=10) | **1.54E-32** | 2.62E-32 | 13.3632 |  |
|  | AH+ (κ=10) | 5.24E-06 | **2.64E-32** | NaN |  |
|  | GP (κ=10) | **2.47E-32** | 2.5721 | NaN |  |
|  | ES (κ=10) | **3.70E-32** | NaN | NaN |  |
|  |  |  |  |  |  |
