# Supplementary material for: Genome-Wide Integration on Transcription Factors, Histone Acetylation and Gene Expression Reveals Genes Co-Regulated by Histone Modification Patterns
Source: PLoS One. 2011 Jul 29;6(7):e22281. doi: 10.1371/journal.pone.0022281 (PMC3146477; doi:10.1371/journal.pone.0022281)
Supplement: Table S2 — Results of preliminary experiment: Variances of the coordinates of cluster centroids obtained by clustering of genes in datasets TR, AH+, GP and ES. We used the parameter set fixed in Table S1 for our approach. Also the variance of our method was obtained by the same manner as that of Table S1. On the other hand, the variance of k-means was obtained in the same manner. That is, we repeated the following process three times: we run k-means 1,000 times with random initial values and obtain the best cluster sets, and computed the variance of the coordinates of cluster centroids over resultant three cluster sets. The smallest value for each dataset is in boldface. This result clearly shows the advantage of our approach over k-means in reproducibility and stability of resultant clusters. (DOC) [file pone.0022281.s004.doc]

**Natsume-Kitatani et al., Table S2**

|  |  | *k*-means | Our method |  |  |
| --- | --- | --- | --- | --- | --- |
|  | TR (κ=10, *k*=10) | 1.7885 | **2.62E-32** |  |  |
|  | AH+ (κ=10, *k*=10) | 1.1262 | **2.64E-32** |  |  |
|  | GP (κ=10, *k*=5) | 0.0149 | **2.47E-32** |  |  |
|  | ES (κ=10, *k*=5) | 0.6953 | **3.70E-32** |  |  |
