# Supplementary material for: Genome-Wide Integration on Transcription Factors, Histone Acetylation and Gene Expression Reveals Genes Co-Regulated by Histone Modification Patterns
Source: PLoS One. 2011 Jul 29;6(7):e22281. doi: 10.1371/journal.pone.0022281 (PMC3146477; doi:10.1371/journal.pone.0022281)
Supplement: Table S3 — Overrepresented MIPS functions in ChIP-chip data [13] . We show Level 1 and 2 of MIPS functions only. P-values represent the probability of finding the observed number of genes with the specified MIPS function under the null hypothesis that the genes were selected at random. (DOC) [file pone.0022281.s005.doc]

**Natsume-Kitatani et al., Table S3**

| cluster 1 (Number of genes: 189) | *p*-value |
| --- | --- |
| 01.04 phosphate metabolism | 0.002141 |
| 01.07 metabolism of vitamins, cofactors, and prosthetic groups | 0.004537 |
| 42.01 cell wall | 0.006097 |
|  |  |
| cluster 2 (Number of genes: 143) |  |
|  |  |
| cluster 3 (Number of genes: 248) |  |
|  |  |
| cluster 4 (Number of genes: 162) | *p*-value |
| 01.02 nitrogen, sulfur and selenium metabolism | 0.003935 |
|  |  |
| cluster 5 (Number of genes: 175) | *p*-value |
| 02.19 metabolism of energy reserves (e.g. glycogen, trehalose) | 0.000172 |
| 12 PROTEIN SYNTHESIS | 1.74E-07 |
| 12.01 ribosome biogenesis | 7.08E-06 |
|  |  |
| cluster 6 (Number of genes: 181) | *p*-value |
| 14.13 protein/peptide degradation | 0.008807 |
|  |  |
| cluster 7 (Number of genes: 170) | *p*-value |
| 01.05 C-compound and carbohydrate metabolism | 0.002532 |
|  |  |
| cluster 8 (Number of genes: 166) | *p*-value |
| 01 METABOLISM | 0.000372 |
| 01.05 C-compound and carbohydrate metabolism | 4.89E-07 |
| 02 ENERGY | 0.000907 |
| 02.01 glycolysis and gluconeogenesis | 0.001016 |
| 02.16 fermentation | 0.008603 |
| 20.01 transported compounds (substrates) | 0.000452 |
| 20.03 transport facilities | 0.000852 |
| 30 CELLULAR COMMUNICATION/SIGNAL TRANSDUCTION MECHANISM | 0.000193 |
| 30.01 cellular signalling | 0.000989 |
| 43 CELL TYPE DIFFERENTIATION | 0.000195 |
| 43.01 fungal/microorganismic cell type differentiation | 0.000195 |
|  |  |
| cluster 9 (Number of genes: 182) | *p*-value |
| 10.01 DNA processing | 0.002547 |
| 11.04 RNA processing | 0.002088 |
| 14 PROTEIN FATE (folding, modification, destination) | 0.00067 |
| 14.07 protein modification | 0.007233 |
| 16 PROTEIN WITH BINDING FUNCTION OR COFACTOR REQUIREMENT (structural or catalytic) | 0.007799 |
|  |  |
| cluster 10 (Number of genes: 140) | *p*-value |
| 01 METABOLISM | 0.006413 |
|  |  |
