# Supplementary material for: Genome-Wide Integration on Transcription Factors, Histone Acetylation and Gene Expression Reveals Genes Co-Regulated by Histone Modification Patterns
Source: PLoS One. 2011 Jul 29;6(7):e22281. doi: 10.1371/journal.pone.0022281 (PMC3146477; doi:10.1371/journal.pone.0022281)
Supplement: Table S4 — Overrepresented MIPS functions in ChIP-chip data [14] . We show Level 1 and 2 of MIPS functions only. P-values represent the probability of finding the observed number of genes with the specified MIPS function under the null hypothesis that the genes were selected at random. (DOC) [file pone.0022281.s006.doc]

**Natsume-Kitatani et al., Table S4**

| cluster 1 (Number of genes: 197) | *p*-value |
| --- | --- |
| 01.04 phosphate metabolism | 0.003712 |
| 12.10 aminoacyl-tRNA-synthetases | 0.000204 |
|  |  |
| cluster 2 (Number of genes: 142) | *p-*value |
| 14 PROTEIN FATE (folding, modification, destination) | 0.003946 |
| 14.01 protein folding and stabilization | 0.000274 |
| 14.13 protein/peptide degradation | 0.006141 |
| 16 PROTEIN WITH BINDING FUNCTION OR COFACTOR REQUIREMENT (structural or catalytic) | 0.000143 |
| 16.01 protein binding | 0.008748 |
| 16.19 nucleotide/nucleoside/nucleobase binding | 0.005735 |
|  |  |
| cluster 3 (Number of genes: 152) | *p*-value |
| 01 METABOLISM | 0.000321 |
| 01.04 phosphate metabolism | 0.007629 |
| 01.05 C-compound and carbohydrate metabolism | 0.000649 |
| 01.07 metabolism of vitamins, cofactors, and prosthetic groups | 0.002521 |
|  |  |
| cluster 4 (Number of genes: 154) | *p*-value |
| 01 METABOLISM | 0.000166 |
| 01.01 amino acid metabolism | 0.007425 |
| 01.05 C-compound and carbohydrate metabolism | 0.000649 |
| 02 ENERGY | 0.000276 |
| 02.07 pentose-phosphate pathway | 0.000263 |
| 02.16 fermentation | 0.00598 |
| 16.21 complex cofactor/cosubstrate/vitamine binding | 0.003041 |
|  |  |
| cluster 5 (Number of genes: 171) |  |
|  |  |
| cluster 6 (Number of genes: 185) | *p-*value |
| 14.04 protein targeting, sorting and translocation | 0.009156 |
| 16.09 lipid binding | 0.001348 |
| 20 CELLULAR TRANSPORT, TRANSPORT FACILITIES AND TRANSPORT ROUTES | 5.84E-05 |
| 20.09 transport routes | 0.001839 |
|  |  |
| cluster 7 (Number of genes: 205) | *p*-value |
| 01.01 amino acid metabolism | 0.002858 |
| 01.02 nitrogen, sulfur and selenium metabolism | 0.004758 |
| 02.07 pentose-phosphate pathway | 0.007499 |
|  |  |
| cluster 8 (Number of genes: 163) | *p-*value |
| 10 CELL CYCLE AND DNA PROCESSING | 0.000477 |
| 10.01 DNA processing | 0.005527 |
| 10.03 cell cycle | 0.003955 |
|  |  |
| cluster 9 (Number of genes: 147) | *p*-value |
| 12 PROTEIN SYNTHESIS | 3.74E-12 |
| 12.01 ribosome biogenesis | 8.29E-13 |
|  |  |
| cluster 10 (Number of genes: 240) | *p*-value |
| 01 METABOLISM | 0.00058 |
| 01.04 phosphate metabolism | 0.000867 |
| 14.07 protein modification | 0.001663 |
| 14.13 protein/peptide degradation | 0.002442 |
| 16.19 nucleotide/nucleoside/nucleobase binding | 0.003092 |
| 20 CELLULAR TRANSPORT, TRANSPORT FACILITIES AND TRANSPORT ROUTES | 0.0064 |
| 20.03 transport facilities | 0.004261 |
| 30 CELLULAR COMMUNICATION/SIGNAL TRANSDUCTION MECHANISM | 3.97E-05 |
| 30.01 cellular signalling | 3.04E-05 |
| 30.05 transmembrane signal transduction | 0.006237 |
| 34 INTERACTION WITH THE ENVIRONMENT | 0.0011 |
| 40 CELL FATE | 0.005095 |
| 40.01 cell growth / morphogenesis | 0.005596 |
| 42.01 cell wall | 0.004301 |
|  |  |
