# Supplementary material for: Genome-Wide Integration on Transcription Factors, Histone Acetylation and Gene Expression Reveals Genes Co-Regulated by Histone Modification Patterns
Source: PLoS One. 2011 Jul 29;6(7):e22281. doi: 10.1371/journal.pone.0022281 (PMC3146477; doi:10.1371/journal.pone.0022281)
Supplement: Table S5 — Overrepresented MIPS functions in microarray data (GSE9217). We show Level 1 and 2 of MIPS functions only. P-values represent the probability of finding the observed number of genes with the specified MIPS function under the null hypothesis that the genes were selected at random. (DOC) [file pone.0022281.s007.doc]

**Natsume-Kitatani et al., Table S5**

| cluster 1 (Number of genes: 363) | *p*-value |
| --- | --- |
| 01 METABOLISM | 0.008379 |
| 16.19 nucleotide/nucleoside/nucleobase binding | 0.003177 |
| 42.04 cytoskeleton/structural proteins | 0.006775 |
|  |  |
| cluster 2 (Number of genes: 465) | *p*-value |
| 16.09 lipid binding | 0.007073 |
| 42.25 vacuole or lysosome | 0.004977 |
|  |  |
| cluster 3 (Number of genes: 274) | *p*-value |
| 01.05 C-compound and carbohydrate metabolism | 9.83E-06 |
| 02 ENERGY | 2.07E-11 |
| 02.10 tricarboxylic-acid pathway (citrate cycle, Krebs cycle, TCA cycle) | 0.00216 |
| 02.11 electron transport and membrane-associated energy conservation | 0.005397 |
| 02.13 respiration | 3.22E-08 |
| 02.19 metabolism of energy reserves (e.g. glycogen, trehalose) | 0.000744 |
| 12 PROTEIN SYNTHESIS | 0.007954 |
| 12.10 aminoacyl-tRNA-synthetases | 0.000264 |
| 16.21 complex cofactor/cosubstrate/vitamine binding | 0.003942 |
| 20.01 transported compounds (substrates) | 0.000641 |
| 42.16 mitochondrion | 1.17E-07 |
|  |  |
| cluster 4 (Number of genes: 184) | *p*-value |
| 01.04 phosphate metabolism | 0.00551 |
| 02.01 glycolysis and gluconeogenesis | 0.007507 |
| 12 PROTEIN SYNTHESIS | 8.51E-06 |
| 12.01 ribosome biogenesis | 0.000286 |
| 14.01 protein folding and stabilization | 0.000379 |
| 32 CELL RESCUE, DEFENSE AND VIRULENCE | 0.000333 |
| 32.01 stress response | 0.000659 |
|  |  |
| cluster 5 (Number of genes: 444) | *p*-value |
| 01 METABOLISM | 0.001085 |
| 01.04 phosphate metabolism | 0.000695 |
| 11 TRANSCRIPTION | 0.004175 |
| 11.04 RNA processing | 0.000991 |
| 14 PROTEIN FATE (folding, modification, destination) | 0.004302 |
| 16.19 nucleotide/nucleoside/nucleobase binding | 0.002941 |
