# Supplementary material for: Genome-Wide Integration on Transcription Factors, Histone Acetylation and Gene Expression Reveals Genes Co-Regulated by Histone Modification Patterns
Source: PLoS One. 2011 Jul 29;6(7):e22281. doi: 10.1371/journal.pone.0022281 (PMC3146477; doi:10.1371/journal.pone.0022281)
Supplement: Table S6 — The result of Tukey-Kramer' multiple comparison test (GSE9217, p <0.05). The lysine residue pairs which were significantly different from each other are listed. (DOC) [file pone.0022281.s008.doc]

**Natsume-Kitatani et al., Table S6**

| **T1H10E1** | | | |
| --- | --- | --- | --- |
| **Lysine residue pairs** | **difference of means** | **wholly significant difference** | ***p*-value** |
| mean[H2A Lys7]=0.21401 vs. mean[H4 Lys16]=0.56039 | diff.= 0.34638 | WSD=0.20116 | *P*=0.00001 |
| mean[H2A Lys7]=0.21401 vs. mean[H3 Lys9]=0.43002 | diff.= 0.21602 | WSD=0.19939 | *P*=0.02235 |
| mean[H3 Lys14]=0.22720 vs. mean[H4 Lys16]=0.56039 | diff.= 0.33319 | WSD=0.19939 | *P*=0.00001 |
| mean[H3 Lys14]=0.22720 vs. mean[H3 Lys9]=0.43002 | diff.= 0.20282 | WSD=0.19740 | *P*=0.03883 |
| mean[H2B Lys11]=0.24695 vs. mean[H4 Lys16]=0.56039 | diff.= 0.31344 | WSD=0.19740 | *P*=0.00005 |
| mean[H2B Lys16]=0.24811 vs. mean[H4 Lys16]=0.56039 | diff.= 0.31228 | WSD=0.19513 | *P*=0.00005 |
| mean[H4 Lys12]=0.26763 vs. mean[H4 Lys16]=0.56039 | diff.= 0.29277 | WSD=0.19249 | *P*=0.00016 |
| mean[H4 Lys8]=0.28115 vs. mean[H4 Lys16]=0.56039 | diff.= 0.27924 | WSD=0.18934 | *P*=0.00034 |
| mean[H3 Lys23]=0.31417 vs. mean[H4 Lys16]=0.56039 | diff.= 0.24622 | WSD=0.18547 | *P*=0.00212 |
| mean[H3 Lys18]=0.31968 vs. mean[H4 Lys16]=0.56039 | diff.= 0.24071 | WSD=0.18043 | *P*=0.00229 |
| mean[H3 Lys27]=0.33113 vs. mean[H4 Lys16]=0.56039 | diff.= 0.22926 | WSD=0.17331 | *P*=0.00311 |
|  |  |  |  |
| **T3H10E1** | | | |
| **Lysine residue pairs** | **difference of means** | **wholly significant difference** | ***p-*value** |
| mean[H3 Lys14]=0.21968 vs. mean[H4 Lys16]=0.53109 | diff.= 0.31141 | WSD=0.18213 | *P*=0.00001 |
| mean[H3 Lys14]=0.21968 vs. mean[H3 Lys9]=0.41626 | diff.= 0.19658 | WSD=0.18053 | *P*=0.02124 |
| mean[H2B Lys16]=0.23740 vs. mean[H4 Lys16]=0.53109 | diff.= 0.29369 | WSD=0.18053 | *P*=0.00003 |
| mean[H2B Lys16]=0.23740 vs. mean[H3 Lys9]=0.41626 | diff.= 0.17886 | WSD=0.17872 | *P*=0.04964 |
| mean[H2B Lys11]=0.23854 vs. mean[H4 Lys16]=0.53109 | diff.= 0.29255 | WSD=0.17872 | *P*=0.00003 |
| mean[H2B Lys11]=0.23854 vs. mean[H3 Lys9]=0.41626 | diff.= 0.17773 | WSD=0.17666 | *P*=0.04739 |
| mean[H2A Lys7]=0.24627 vs. mean[H4 Lys16]=0.53109 | diff.= 0.28482 | WSD=0.17666 | *P*=0.00004 |
| mean[H4 Lys12]=0.26496 vs. mean[H4 Lys16]=0.53109 | diff.= 0.26613 | WSD=0.17427 | *P*=0.00016 |
| mean[H3 Lys23]=0.27551 vs. mean[H4 Lys16]=0.53109 | diff.= 0.25558 | WSD=0.17141 | *P*=0.00029 |
| mean[H4 Lys8]=0.27675 vs. mean[H4 Lys16]=0.53109 | diff.= 0.25435 | WSD=0.16789 | *P*=0.00026 |
| mean[H3 Lys27]=0.29121 vs. mean[H4 Lys16]=0.53109 | diff.= 0.23988 | WSD=0.16333 | *P*=0.00055 |
| mean[H3 Lys18]=0.32511 vs. mean[H4 Lys16]=0.53109 | diff.= 0.20598 | WSD=0.15688 | *P*=0.00348 |
|  |  |  |  |
| **T6H10E1** | | | |
| **Lysine residue pairs** | **difference of means** | **wholly significant difference** | ***p-*value** |
| mean[H2B Lys16]=0.23374 vs. mean[H4 Lys16]=0.56000 | diff.= 0.32626 | WSD=0.20011 | *P*=0.00002 |
| mean[H2B Lys11]=0.24051 vs. mean[H4 Lys16]=0.56000 | diff.= 0.31949 | WSD=0.19835 | *P*=0.00004 |
| mean[H3 Lys14]=0.25190 vs. mean[H4 Lys16]=0.56000 | diff.= 0.30810 | WSD=0.19636 | *P*=0.00007 |
| mean[H2A Lys7]=0.25329 vs. mean[H4 Lys16]=0.56000 | diff.= 0.30671 | WSD=0.19410 | *P*=0.00007 |
| mean[H3 Lys23]=0.28770 vs. mean[H4 Lys16]=0.56000 | diff.= 0.27230 | WSD=0.19147 | *P*=0.00058 |
| mean[H4 Lys8]=0.30363 vs. mean[H4 Lys16]=0.56000 | diff.= 0.25637 | WSD=0.18833 | *P*=0.00133 |
| mean[H4 Lys12]=0.30869 vs. mean[H4 Lys16]=0.56000 | diff.= 0.25131 | WSD=0.18447 | *P*=0.00149 |
| mean[H3 Lys18]=0.32708 vs. mean[H4 Lys16]=0.56000 | diff.= 0.23292 | WSD=0.17945 | *P*=0.00338 |
| mean[H3 Lys27]=0.32795 vs. mean[H4 Lys16]=0.56000 | diff.= 0.23205 | WSD=0.17236 | *P*=0.00252 |
| mean[H3 Lys9]=0.39176 vs. mean[H4 Lys16]=0.56000 | diff.= 0.16824 | WSD=0.16037 | *P*=0.03585 |
|  |  |  |  |
| **T5H9E4** | | | |
| **Lysine residue pairs** | **difference of means** | **wholly significant difference** | ***p*-value** |
| mean[H2B Lys16]=0.60296 vs. mean[H3 Lys18]=0.97233 | diff.= 0.36937 | WSD=0.19810 | *P*=0.00000 |
| mean[H2B Lys16]=0.60296 vs. mean[H3 Lys27]=0.96546 | diff.= 0.36250 | WSD=0.19639 | *P*=0.00000 |
| mean[H2B Lys11]=0.63619 vs. mean[H3 Lys18]=0.97233 | diff.= 0.33613 | WSD=0.19639 | *P*=0.00000 |
| mean[H2B Lys16]=0.60296 vs. mean[H3 Lys14]=0.94658 | diff.= 0.34362 | WSD=0.19445 | *P*=0.00000 |
| mean[H2B Lys11]=0.63619 vs. mean[H3 Lys27]=0.96546 | diff.= 0.32927 | WSD=0.19445 | *P*=0.00001 |
| mean[H4 Lys16]=0.69398 vs. mean[H3 Lys18]=0.97233 | diff.= 0.27834 | WSD=0.19445 | *P*=0.00033 |
| mean[H2B Lys16]=0.60296 vs. mean[H3 Lys9]=0.93650 | diff.= 0.33354 | WSD=0.19224 | *P*=0.00000 |
| mean[H2B Lys11]=0.63619 vs. mean[H3 Lys14]=0.94658 | diff.= 0.31039 | WSD=0.19224 | *P*=0.00003 |
| mean[H4 Lys16]=0.69398 vs. mean[H3 Lys27]=0.96546 | diff.= 0.27147 | WSD=0.19224 | *P*=0.00047 |
| mean[H4 Lys8]=0.77091 vs. mean[H3 Lys18]=0.97233 | diff.= 0.20142 | WSD=0.19224 | *P*=0.03194 |
| mean[H2B Lys16]=0.60296 vs. mean[H3 Lys23]=0.93617 | diff.= 0.33321 | WSD=0.18967 | *P*=0.00000 |
| mean[H2B Lys11]=0.63619 vs. mean[H3 Lys9]=0.93650 | diff.= 0.30030 | WSD=0.18967 | *P*=0.00005 |
| mean[H4 Lys16]=0.69398 vs. mean[H3 Lys14]=0.94658 | diff.= 0.25260 | WSD=0.18967 | *P*=0.00145 |
| mean[H4 Lys8]=0.77091 vs. mean[H3 Lys27]=0.96546 | diff.= 0.19455 | WSD=0.18967 | *P*=0.03961 |
| mean[H2B Lys16]=0.60296 vs. mean[H4 Lys12]=0.88995 | diff.= 0.28699 | WSD=0.18661 | *P*=0.00012 |
| mean[H2B Lys11]=0.63619 vs. mean[H3 Lys23]=0.93617 | diff.= 0.29997 | WSD=0.18661 | *P*=0.00004 |
| mean[H4 Lys16]=0.69398 vs. mean[H3 Lys9]=0.93650 | diff.= 0.24251 | WSD=0.18661 | *P*=0.00234 |
| mean[H2B Lys16]=0.60296 vs. mean[H2A Lys7]=0.83870 | diff.= 0.23574 | WSD=0.18282 | *P*=0.00291 |
| mean[H2B Lys11]=0.63619 vs. mean[H4 Lys12]=0.88995 | diff.= 0.25376 | WSD=0.18282 | *P*=0.00093 |
| mean[H4 Lys16]=0.69398 vs. mean[H3 Lys23]=0.93617 | diff.= 0.24218 | WSD=0.18282 | *P*=0.00196 |
| mean[H2B Lys11]=0.63619 vs. mean[H2A Lys7]=0.83870 | diff.= 0.20251 | WSD=0.17790 | *P*=0.01501 |
| mean[H4 Lys16]=0.69398 vs. mean[H4 Lys12]=0.88995 | diff.= 0.19597 | WSD=0.17790 | *P*=0.02103 |
