# Supplementary material for: Genome-Wide Integration on Transcription Factors, Histone Acetylation and Gene Expression Reveals Genes Co-Regulated by Histone Modification Patterns
Source: PLoS One. 2011 Jul 29;6(7):e22281. doi: 10.1371/journal.pone.0022281 (PMC3146477; doi:10.1371/journal.pone.0022281)
Supplement: Table S7 — Overrepresented MIPS functions in microarray data (GSE9840). We show Level 1 and 2 of MIPS functions only. P-values represent the probability of finding the observed number of genes with the specified MIPS function under the null hypothesis that the genes were selected at random. (DOC) [file pone.0022281.s009.doc]

**Natsume-Kitatani et al., Table S7**

| cluster 1 (Number of genes: 514) | *p*-value |
| --- | --- |
| 14 PROTEIN FATE (folding, modification, destination) | 4.89E-05 |
| 14.07 protein modification | 0.001559 |
| 14.13 protein/peptide degradation | 0.007483 |
| 32 CELL RESCUE, DEFENSE AND VIRULENCE | 0.005849 |
| 32.05 disease, virulence and defense | 0.006995 |
| 34 INTERACTION WITH THE ENVIRONMENT | 0.009886 |
|  |  |
| cluster 2 (Number of genes: 255) | *p*-value |
| 11 TRANSCRIPTION | 0.003657 |
| 11.04 RNA processing | 7.72E-05 |
| 11.06 RNA modification | 0.001073 |
| 12 PROTEIN SYNTHESIS | 9.69E-11 |
| 12.01 ribosome biogenesis | 2.26E-09 |
| 12.10 aminoacyl-tRNA-synthetases | 0.004645 |
| 16.03 nucleic acid binding | 0.006208 |
|  |  |
| cluster 3 (Number of genes: 352) | *p*-value |
| 01.04 phosphate metabolism | 0.004315 |
| 10 CELL CYCLE AND DNA PROCESSING | 2.24E-05 |
| 10.01 DNA processing | 0.0026 |
| 10.03 cell cycle | 2.50E-05 |
| 11.06 RNA modification | 0.009699 |
| 16.19 nucleotide/nucleoside/nucleobase binding | 0.008691 |
| 18.01 regulation by | 0.004264 |
|  |  |
| cluster 4 (Number of genes: 347) | *p*-value |
| 01 METABOLISM | 2.26E-06 |
| 01.01 amino acid metabolism | 0.000505 |
| 01.04 phosphate metabolism | 0.002066 |
| 01.05 C-compound and carbohydrate metabolism | 0.000355 |
| 02.01 glycolysis and gluconeogenesis | 0.001515 |
| 12.04 translation | 0.000362 |
| 12.10 aminoacyl-tRNA-synthetases | 0.000245 |
| 16.21 complex cofactor/cosubstrate/vitamine binding | 0.001323 |
| 20 CELLULAR TRANSPORT, TRANSPORT FACILITIES AND TRANSPORT ROUTES | 0.000349 |
| 20.01 transported compounds (substrates) | 0.001614 |
| 20.09 transport routes | 0.001322 |
| 30 CELLULAR COMMUNICATION/SIGNAL TRANSDUCTION MECHANISM | 0.00306 |
| 30.01 cellular signalling | 0.008252 |
| 32 CELL RESCUE, DEFENSE AND VIRULENCE | 0.00261 |
| 32.07 detoxification | 0.002257 |
|  |  |
| cluster 5 (Number of genes: 262) | *p*-value |
| 01 METABOLISM | 1.70E-05 |
| 01.05 C-compound and carbohydrate metabolism | 0.000148 |
| 01.06 lipid, fatty acid and isoprenoid metabolism | 0.009486 |
| 02 ENERGY | 4.03E-06 |
| 02.10 tricarboxylic-acid pathway (citrate cycle, Krebs cycle, TCA cycle) | 3.17E-05 |
| 02.16 fermentation | 0.003342 |
| 02.25 oxidation of fatty acids | 0.005238 |
| 20 CELLULAR TRANSPORT, TRANSPORT FACILITIES AND TRANSPORT ROUTES | 0.00081 |
| 20.01 transported compounds (substrates) | 0.00471 |
