# Supplementary material for: Genome-Wide Integration on Transcription Factors, Histone Acetylation and Gene Expression Reveals Genes Co-Regulated by Histone Modification Patterns
Source: PLoS One. 2011 Jul 29;6(7):e22281. doi: 10.1371/journal.pone.0022281 (PMC3146477; doi:10.1371/journal.pone.0022281)
Supplement: Table S8 — The result of Tukey-Kramer' multiple comparison test (GSE9840, p <0.05). The lysine residue pairs which were significantly different from each other are listed. (DOC) [file pone.0022281.s010.doc]

**Natsume-Kitatani et al., Table S8**

| **T3H10E3** | | | |
| --- | --- | --- | --- |
| **Lysine residue pairs** | **difference of means** | **wholly significant difference** | ***p*-value** |
| mean[H4 Lys12]=0.20353 vs. mean[H4 Lys16]=0.43273 | diff.= 0.22920 | WSD=0.19812 | *P*=0.01013 |
| mean[H3 Lys14]=0.20671 vs. mean[H4 Lys16]=0.43273 | diff.= 0.22603 | WSD=0.19638 | *P*=0.01104 |
| mean[H4 Lys8]=0.23496 vs. mean[H4 Lys16]=0.43273 | diff.= 0.19778 | WSD=0.19442 | *P*=0.04270 |
| mean[H2A Lys7]=0.23564 vs. mean[H4 Lys16]=0.43273 | diff.= 0.19709 | WSD=0.19219 | *P*=0.03971 |
| mean[H2B Lys16]=0.24096 vs. mean[H4 Lys16]=0.43273 | diff.= 0.19178 | WSD=0.18959 | *P*=0.04519 |
| mean[H2B Lys11]=0.24321 vs. mean[H4 Lys16]=0.43273 | diff.= 0.18953 | WSD=0.18649 | *P*=0.04348 |
|  |  |  |  |
| **T9H1E5** | | | |
| **Lysine residue pairs** | **difference of means** | **wholly significant difference** | ***p-*value** |
| mean[H4 Lys16]=0.23928 vs. mean[H2B Lys16]=0.54097 | diff.= 0.30169 | WSD=0.23687 | *P*=0.00286 |
| mean[H4 Lys12]=0.25244 vs. mean[H2B Lys16]=0.54097 | diff.= 0.28852 | WSD=0.23476 | *P*=0.00493 |
| mean[H4 Lys8]=0.26937 vs. mean[H2B Lys16]=0.54097 | diff.= 0.27160 | WSD=0.23240 | *P*=0.00974 |
| mean[H3 Lys14]=0.30702 vs. mean[H2B Lys16]=0.54097 | diff.= 0.23394 | WSD=0.22970 | *P*=0.04248 |
| mean[H3 Lys27]=0.31340 vs. mean[H2B Lys16]=0.54097 | diff.= 0.22757 | WSD=0.22657 | *P*=0.04815 |
|  |  |  |  |
| **T5H9E2** | | | |
| **Lysine residue pairs** | **difference of means** | **wholly significant difference** | ***p-*value** |
| mean[H2B Lys16]=0.56880 vs. mean[H3 Lys18]=0.94114 | diff.= 0.37234 | WSD=0.21003 | *P*=0.00000 |
| mean[H2B Lys16]=0.56880 vs. mean[H3 Lys27]=0.94033 | diff.= 0.37154 | WSD=0.20821 | *P*=0.00000 |
| mean[H2B Lys11]=0.58831 vs. mean[H3 Lys18]=0.94114 | diff.= 0.35283 | WSD=0.20821 | *P*=0.00001 |
| mean[H2B Lys16]=0.56880 vs. mean[H3 Lys23]=0.92208 | diff.= 0.35329 | WSD=0.20616 | *P*=0.00000 |
| mean[H2B Lys11]=0.58831 vs. mean[H3 Lys27]=0.94033 | diff.= 0.35203 | WSD=0.20616 | *P*=0.00001 |
| mean[H4 Lys16]=0.66568 vs. mean[H3 Lys18]=0.94114 | diff.= 0.27545 | WSD=0.20616 | *P*=0.00115 |
| mean[H2B Lys16]=0.56880 vs. mean[H3 Lys14]=0.91700 | diff.= 0.34820 | WSD=0.20383 | *P*=0.00001 |
| mean[H2B Lys11]=0.58831 vs. mean[H3 Lys23]=0.92208 | diff.= 0.33378 | WSD=0.20383 | *P*=0.00002 |
| mean[H4 Lys16]=0.66568 vs. mean[H3 Lys27]=0.94033 | diff.= 0.27465 | WSD=0.20383 | *P*=0.00107 |
| mean[H4 Lys8]=0.73112 vs. mean[H3 Lys18]=0.94114 | diff.= 0.21002 | WSD=0.20383 | *P*=0.03768 |
| mean[H2B Lys16]=0.56880 vs. mean[H3 Lys9]=0.90255 | diff.= 0.33375 | WSD=0.20111 | *P*=0.00002 |
| mean[H2B Lys11]=0.58831 vs. mean[H3 Lys14]=0.91700 | diff.= 0.32869 | WSD=0.20111 | *P*=0.00002 |
| mean[H4 Lys16]=0.66568 vs. mean[H3 Lys23]=0.92208 | diff.= 0.25640 | WSD=0.20111 | *P*=0.00283 |
| mean[H4 Lys8]=0.73112 vs. mean[H3 Lys27]=0.94033 | diff.= 0.20921 | WSD=0.20111 | *P*=0.03454 |
| mean[H2B Lys16]=0.56880 vs. mean[H4 Lys12]=0.85433 | diff.= 0.28553 | WSD=0.19786 | *P*=0.00039 |
| mean[H2B Lys11]=0.58831 vs. mean[H3 Lys9]=0.90255 | diff.= 0.31424 | WSD=0.19786 | *P*=0.00006 |
| mean[H4 Lys16]=0.66568 vs. mean[H3 Lys14]=0.91700 | diff.= 0.25132 | WSD=0.19786 | *P*=0.00324 |
| mean[H2B Lys16]=0.56880 vs. mean[H2A Lys7]=0.79467 | diff.= 0.22587 | WSD=0.19385 | *P*=0.01081 |
| mean[H2B Lys11]=0.58831 vs. mean[H4 Lys12]=0.85433 | diff.= 0.26602 | WSD=0.19385 | *P*=0.00111 |
| mean[H4 Lys16]=0.66568 vs. mean[H3 Lys9]=0.90255 | diff.= 0.23687 | WSD=0.19385 | *P*=0.00602 |
| mean[H2B Lys11]=0.58831 vs. mean[H2A Lys7]=0.79467 | diff.= 0.20636 | WSD=0.18863 | *P*=0.02245 |
| mean[H4 Lys16]=0.66568 vs. mean[H4 Lys12]=0.85433 | diff.= 0.18864 | WSD=0.18863 | *P*=0.04998 |
